# Supplementary material for: Genes Modulating Butyrate Metabolism for Assessing Clinical Prognosis and Responses to Systematic Therapies in Hepatocellular Carcinoma
Source: Biomolecules. 2022 Dec 27;13(1):52. doi: 10.3390/biom13010052 (PMC9856074; doi:10.3390/biom13010052)
Supplement: Supplementary file 1 [file biomolecules-13-00052-s001.zip › biomolecules-2082425-supplementary.pdf]

Supplementary material

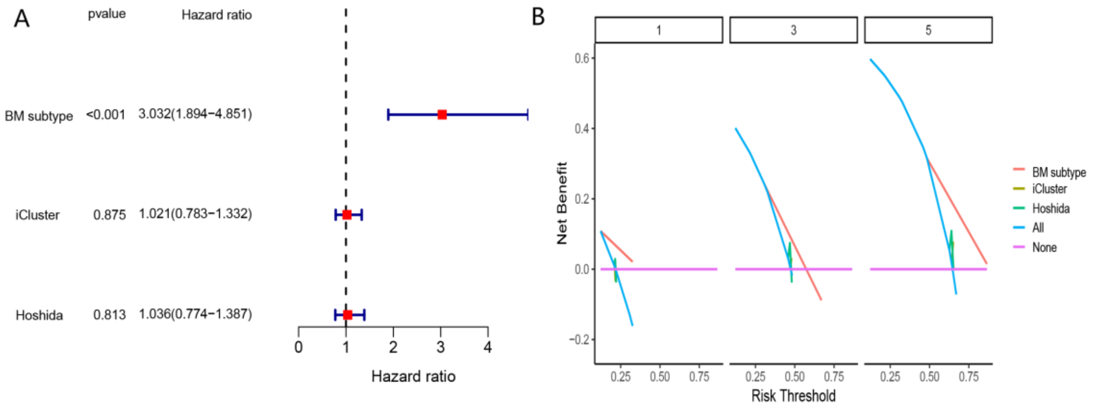

Figure S1.

A. Univariate Cox analysis of BM subtype, iCluster, and Hoshida subtype. B. Decision curve analysis of BM subtype, iCluster, and Hoshida subtype for prediction of prognosis of HCC.

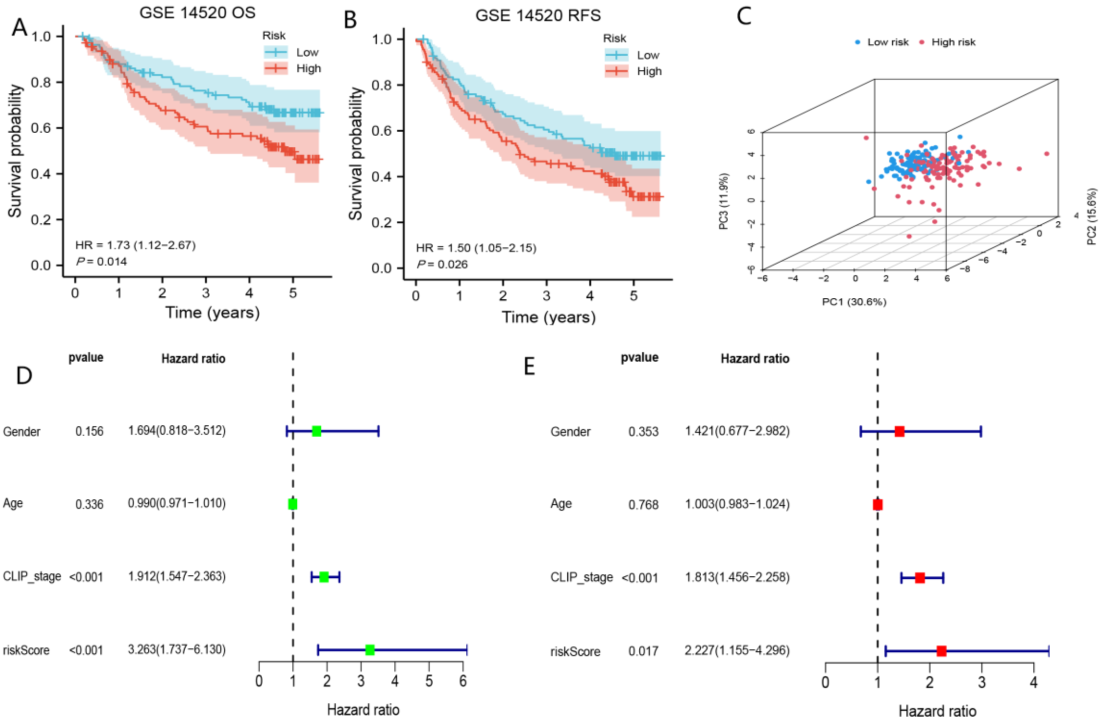

Figure S2. The predictive performances of BMGs in GSE14520 cohort. (A) KM curve of OS. (B) KM curve of recurrence free survival (RFS). (C) PCA plot. (D) Univariate Cox analysis of risk score, TNM-stage, age, and gender. (E) Multivariate Cox analysis of risk score and TNM-satge.

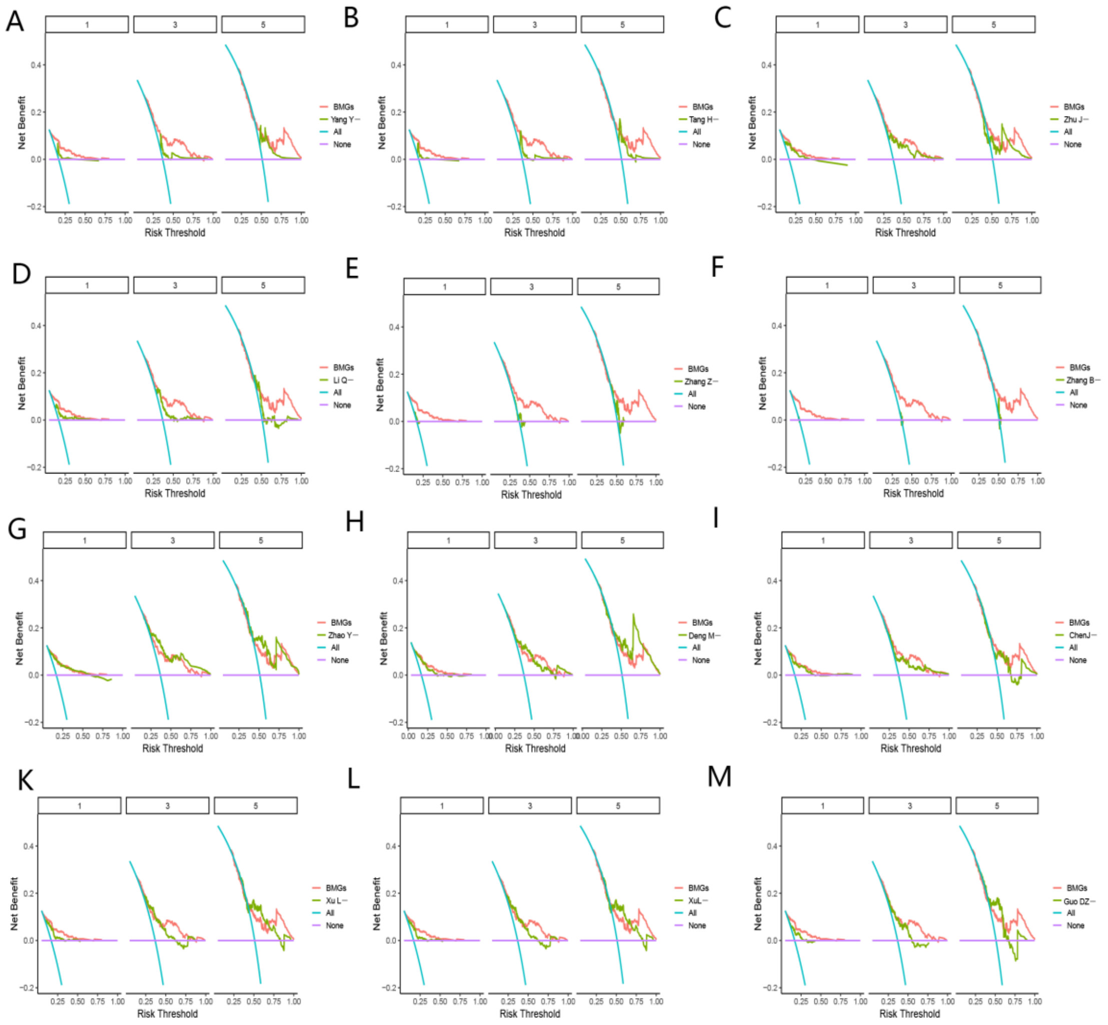

Figure S3. Comparison of BMGs with other 12 gene signatures with the use of DCA.

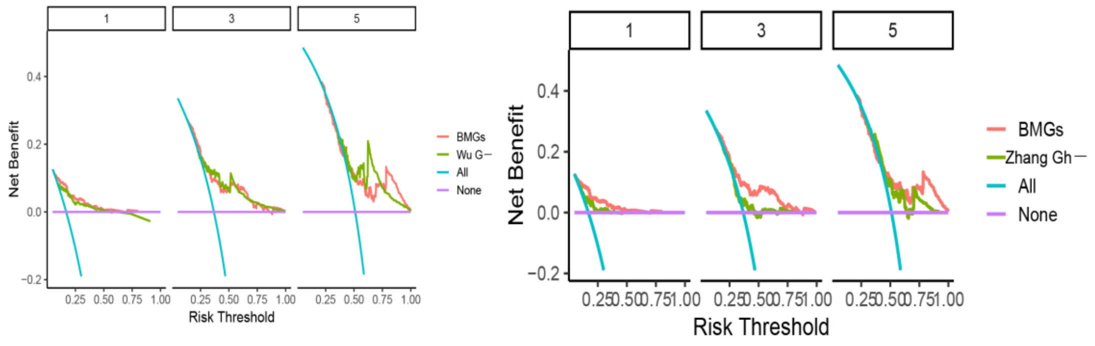

Figure S4. Comparison of BMGs with other 2 gene signatures with the use of DCA.

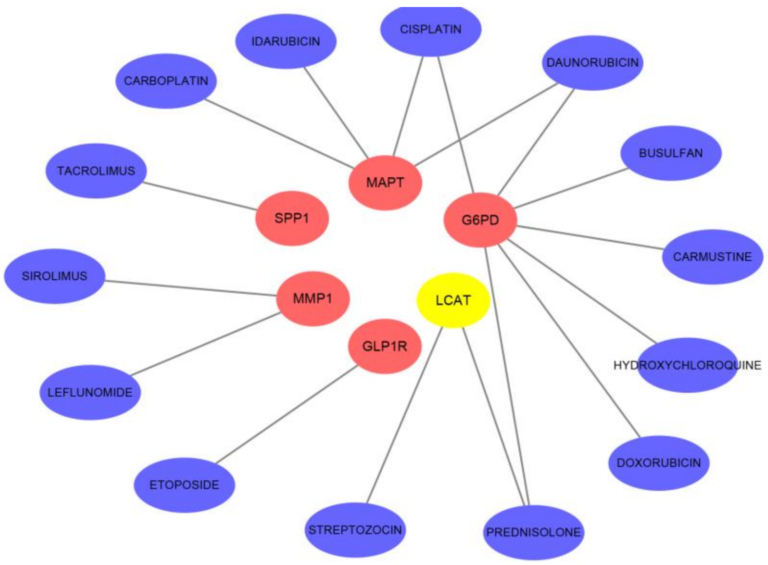

Figure S5. Drugs interacting with the six genes comprising the BMGs.

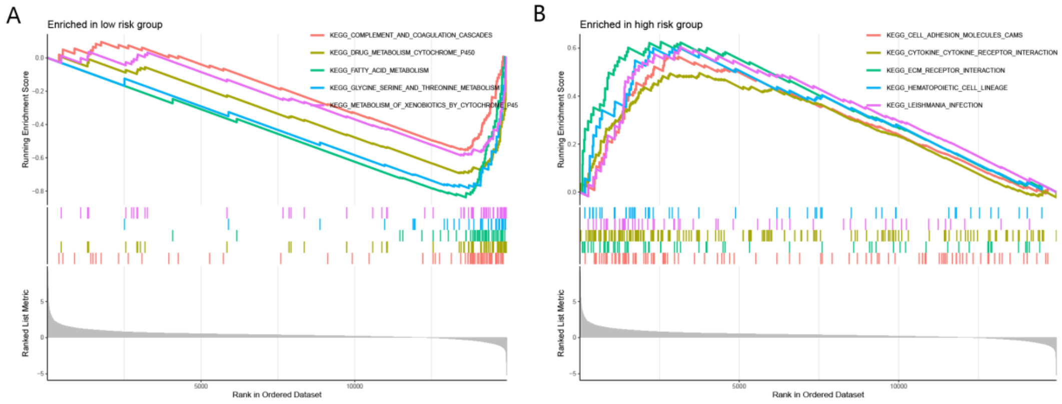

Figure S6 (A).The enriched molecular functions in low-risk group. (B) The enriched molecular functions in high-risk group

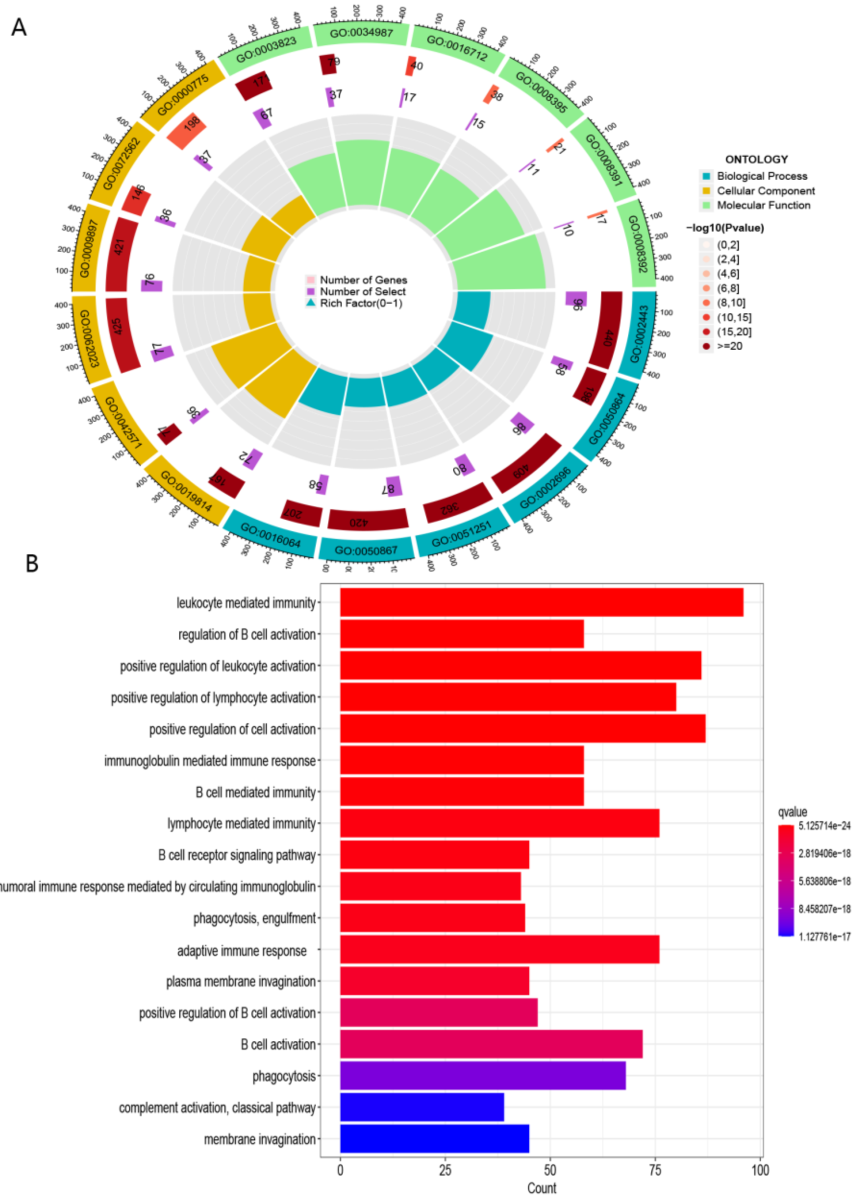

Figure S7. The GO and KEGG analysis of DEGs beteween low- and high-risk patients. (A) GO analysis of DEGs. (B) KEGG analysis of DEGs.

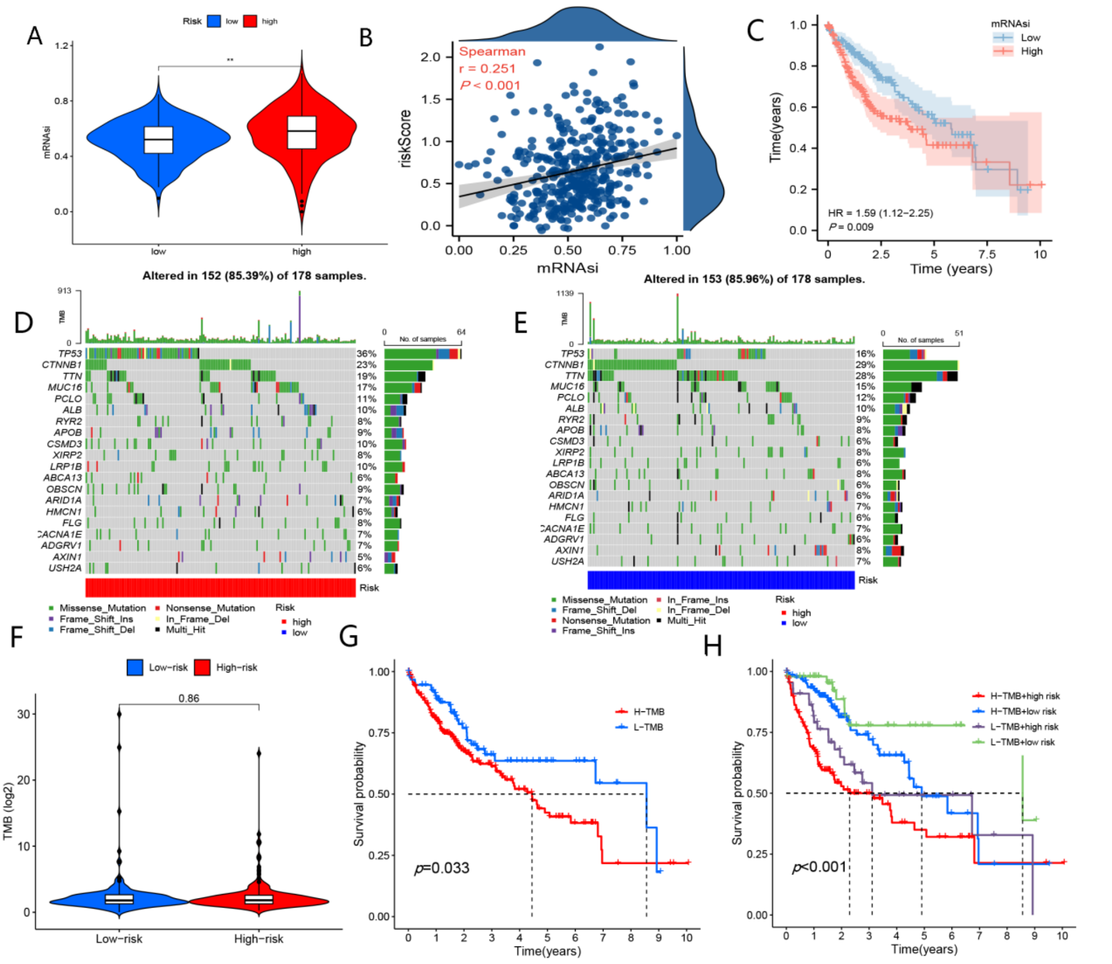

Figure S8. Stemness indices and somatic mutation analysis. (A) Comparison of mRNAsi in high- and low-risk groups. (B) The correlation between risk score and mRNAsi. (C) KM curve of OS for patients in low- and high-mRNAsi group. (D) The top 20 mutated genes in high-risk group. (E) The top 20 mutated genes in low-risk group. (F) Comparison of TMB in high- and low-risk groups. (G) KM curve of TMB. (H) KM curve of TMB+BMGs. ("**",represent no , P<0.01.)
